# Selection and inhibition mechanisms for human voluntary action decisions

**DOI:** 10.1016/j.neuroimage.2012.06.058

**Published:** 2012-10-15

**Authors:** Jiaxiang Zhang, Laura E. Hughes, James B. Rowe

**Affiliations:** a Cognition and Brain Sciences Unit, Medical Research Council, Cambridge CB2 7EF, UK; b Department of Clinical Neurosciences, University of Cambridge, Cambridge CB2 2QQ, UK

**Keywords:** Decision making, Inhibition, Volition, Accumulation, fMRI, Modeling

## Abstract

One can choose between action alternatives that have no apparent difference in their outcomes. Such voluntary action decisions are associated with widespread frontal–parietal activation, and a tendency to inhibit the repetition of a previous action. However, the mechanism of initiating voluntary actions and the functions of different brain regions during this process remains largely unknown. Here, we combine computational modeling and functional magnetic resonance imaging to test the selection and inhibition mechanisms that mediate trial-to-trial voluntary action decisions. We fitted an optimized accumulator model to behavioral responses in a finger-tapping task in which participants were instructed to make chosen actions or specified actions. Model parameters derived from each individual were then applied to estimate the expected accumulated metabolic activity (EAA) engaged in every single trial. The EAA was associated with blood oxygenation level-dependent responses in a decision work that was maximal in the supplementary motor area and the caudal anterior cingulate cortex, consistent with a competitive accumulation-to-threshold mechanism for action decision by these regions. Furthermore, specific inhibition of the previous action's accumulator was related to the suppression of response repetition. This action-specific inhibition correlated with the activity of the right inferior frontal gyrus, when the option to repeat existed. Our findings suggest that human voluntary action decisions are mediated by complementary processes of intentional selection and inhibition.

## Highlights

► An accumulation-to-threshold mechanism supports human voluntary action decisions. ► The decision network is maximal in the medial frontal cortex. ► Suppression of repetition is mediated by the right inferior frontal gyrus.

## Introduction

Deciding between alternative actions sometimes requires extracting meaningful information from noisy sensory signals, a process referred to as perceptual decision making (Gold and Shadlen, 2001). Converging evidence from primate neurophysiology (Mazurek et al., 2003; Roitman and Shadlen, 2002; Shadlen and Newsome, 2001), neuroimaging (Forstmann et al., 2010a; Heekeren et al., 2008; Ploran et al., 2007), behavioral studies (Ratcliff and Smith, 2004), and psychological models (Brown and Heathcote, 2008; Busemeyer and Townsend, 1993; Ratcliff and McKoon, 2008; Usher and McClelland, 2001) suggests that the brain implements an accumulation-to-threshold mechanism during perceptual decision making. Sensory evidence supporting each action is accumulated over time, and the action is committed to when the accumulated information reaches a threshold (Gold and Shadlen, 2007).

People can also voluntarily select their actions when the choice of which action to make is not explicitly guided by noisy sensory attributes or by differential action outcomes. This type of decision (hereafter referred to as voluntary action decision) involves the formation of intentions (Libet, 1985). Previous studies have shown that generating sequential voluntary actions deviates from purely random behavior, for example people tend to avoid repetition of responses over consecutive actions (Baddeley et al., 1998). Neuroimaging studies of voluntary action decisions further indicate that intentional selection of actions involves the medial–frontal cortex (Cunnington et al., 2002; Lau et al., 2004; Rowe et al., 2010; Rushworth et al., 2004). However, the mechanisms of selection between action alternatives and the inhibition of response repetition remain poorly understood.

**Fig. 1:**
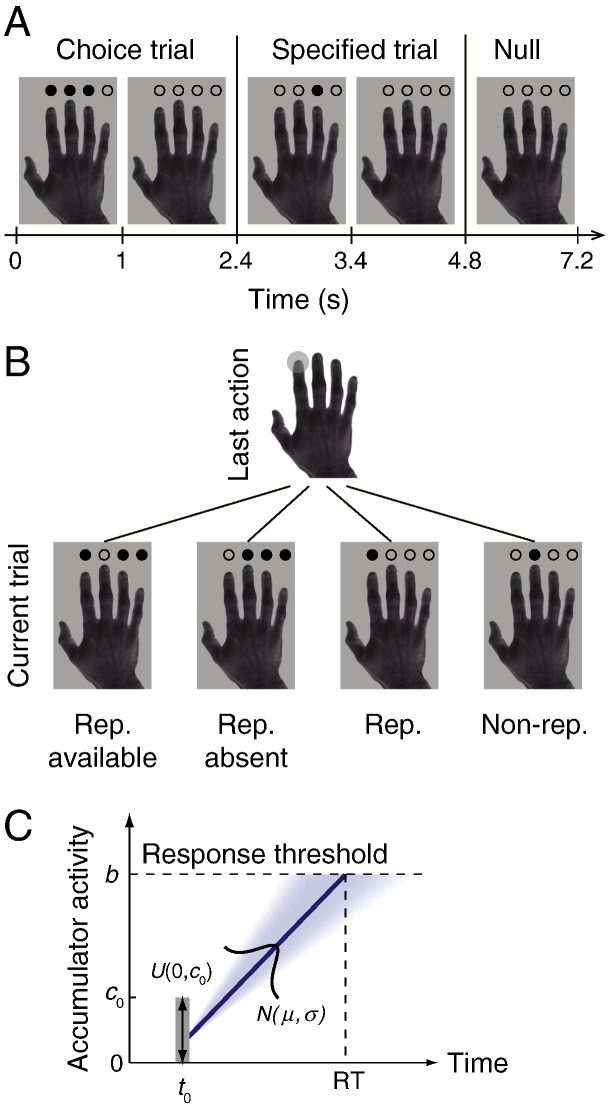
Experiment design and the accumulator model. (A) Examples of trials in the finger-tapping task. Valid actions in each trial were indicated by opaque circles above the corresponding figures. In choice action trials, participants responded with any one of the three valid actions. In specified action trials, participants required to respond with a specified action, indicated by a single opaque circle. (B) Given the response in the last action trial (e.g., the index finger), the repetition alternative was available in half of the trials and absent in other half of the trials. This was implemented in both choice and specified trials. (C) Exemplar time course of accumulator activation in the LBA model. The starting activation of the accumulator is from a uniform distribution. The accumulator activation is linearly accumulated over time with a constant accumulation rate sampled from a normal distribution on each trial, until a response threshold is reached.

Here, we reasoned that during voluntary action decisions the brain accumulates the intention of selecting each action until a threshold is reached, a mechanism analogous to that of evidence accumulation during perceptual decision making. We combined formal computational modeling and fMRI measurements to compare potential accumulation mechanisms for action decisions. Healthy participants voluntarily selected any one of three alternative actions or responded with a specified action in a finger-tapping experiment (Fig. 1A). We fitted accumulator models to individual behavioral data and used the most likely of a large set of models to predict the trial by trial induction of the BOLD response. Our results revealed a decision network, maximal in medial frontal cortex, implementing an accumulation-to-threshold mechanism.

By manipulating the range of alternative choices over successive trials (Fig. 1B), we investigated the mechanism of inhibition of response repetition. We asked first what aspect of the action decision process is modulated over successive choices: a change in response threshold (Forstmann et al., 2010b), the inhibition of accumulation rate (Ludwig et al., 2009), or a combination of the two (Farrell et al., 2010). Second, we asked whether the bias against response repetition arises from processes within the medial–frontal cortex; refractory motor cortical representations; or modulatory inputs from other cortical regions. By exploiting individual differences in the model parameters, we identified brain regions associated with modulatory inhibitory influences, arising external to the network for competitive selection.

## Material and methods

### Participants

Eighteen participants participated in the study (5 males; age range, 20–39 years; mean age, 27 years). All participants were right-handed and none had a history of significant neurological or psychiatric illness. None of the participants had previous experience with the task. The data from two participants were excluded from further analysis because they failed to respond on more than 35% of all trials. The participants gave written informed consent and were paid for their participation. The study was approved by the local research ethics committee.

### Task

Participants performed a visually paced right-hand finger-tapping task adapted from our previous studies (Hughes et al., 2010, in press; Rowe et al., 2010). They were presented with an image of a right hand (4.19° × 6.31°) and pressed a button with one of their four right hand fingers. Four small circles (0.39°) superimposed above the four fingers in the image served as task cues in two trial types: ‘specified action’ and ‘choice action’.

On a *specified action* trial, the task cue contained a single opaque circle indicating which finger to press. On a *choice action* trial, *three* of the four circles in the task cue appeared opaque, indicating that participants could voluntarily select any one of the three fingers to press. For choice action trials, participants were not asked to make “random” choices, because previous research suggests that when participants are asked to generate random choices, they are more inclined to monitor their previous response history (Jahanshahi and Dirnberger, 1999; Mueller et al., 2007; van Eimeren et al., 2006). Instead, participants were asked to make a fresh choice on each trial, regardless of what they had done in previous trials. We did not encourage or discourage repetitions or any particular action sequence. Participants were allowed to repeat the same action made in the previous trial if this choice is available. However, this does not guarantee that some participants do not infer randomness as a goal, but it is likely to promote participants to select between valid actions within each trial.

On each trial the task cue was presented for 1 s, after which the task cue was changed to transparent circles, and the trial duration was 2.4 s (Fig. 1A). Participants were free to make a response at any time during the trial. Although there was no emphasis on the speed of the response, in all conditions participants made responses within 550–650 ms after task cue onset, suggesting that rapid actions in the present study are unlikely associated with high-level or complex proactive strategies. To estimate the BOLD response change between task conditions, we varied the stimulus onset asynchrony between task trials by including ‘null events’ (Friston et al., 1999), in which four transparent circles were continuously presented above the hand image for 2.4 s and no action was required (i.e., simply an extended inter-trial interval from the participant's perspective). The hand image and the four empty circles were presented on the screen at all times throughout the experiment. The experiment comprised a total of 1008 randomly intermixed trials with 50% choice action and 25% specified action trials, interspersed by 25% null events. There were no more than four consecutive trials of the same trial type.

In order to investigate the effect of the previous response history on current trials, we manipulated the range of alternative choices over successive action trials (Fig. 1B). For choice action trials, the option to repeat the last action (e.g., the index finger in Fig. 1B) was available in half of the trials (e.g., repeating the index finger) and not available in other half of the trials (e.g., the index finger is not available), hereafter referred to as “repetition-available” and “repetition-absent” conditions. Similarly for specified action trials, the participants were cued to repeat the last action in half of the trials and they were required to switch to a different action in other half of the trials (e.g., the middle finger in Fig. 1B), hereafter referred to as “repetition” and “non-repetition” conditions. In all conditions, we did not differentiate the type of the last action trial, i.e., the last action trial could either be a choice or specified action trial.

### Accumulator model of action decisions

**Fig. 2:**
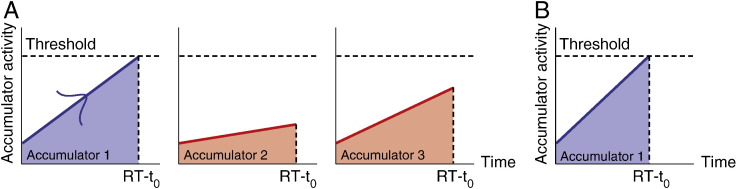
Expected accumulated activity (EAA). (A) In a choice trial with three valid actions, three corresponding accumulators linearly increase their activity over time until the first accumulator reaches the threshold, by which the activities of the two losing accumulators are still lower than the threshold. The predicted EAA is calculated by the summed integral of the accumulator activities prior to the response, as represented by the areas under each line. (B) The EAA in a specified trial. Since only one alternative is valid the EAA of the trial is equivalent to the integral of the activity of the single accumulator.

Behavioral data were analyzed using variants of a cognitive model, the linear ballistic accumulator (LBA) model (Brown and Heathcote, 2008). The LBA model is a simplified instance of sequential sampling models of choice response time (Bogacz et al., 2006; Ratcliff and Smith, 2004), and has been applied to study the neural correlates of speed–accuracy tradeoffs (Forstmann et al., 2010b) and task difficulty (Ho et al., 2009). The model assumes that the decision of when and which action to select is governed by a ‘horse race’ competition among independent accumulators. Each accumulator linearly integrates the evidence (intention) over time in favor of one action, and the choice is selected when the accumulated evidence reaches threshold (Figs. 1C and 2).

We modeled four actions (i.e., four fingers) by independent accumulators *i* (*i* = 1, 2, 3, 4). On a choice trial, three accumulators (corresponding to the three valid actions) are activated with starting activations independently drawn from a uniform distribution [0, *c*_0_]. The activation of each accumulator increases linearly over time with an accumulation rate drawn from an independent normal distribution with mean *μ*_*i*_ and standard deviation *σ*_*i*_. The accumulation process terminates when activation of any accumulator reaches a response threshold *b* (*b* > 0) and a response is triggered by the wining accumulator. On a specified trial, only one accumulator associated with the specified action is activated. The predicted response time (RT) is given by the duration of the accumulation process, plus a constant non-decision time *t*_0_, which may represent the latency associated with stimulus encoding or motor response initiation (Brown and Heathcote, 2008).

The application of the LBA model serves several purposes in the present study. First, the model was used to fit individual RT distributions and choice probabilities. Second, the simplicity and tractability of the LBA model make it possible to investigate the neural correlates of the accumulation mechanism in a trial-by-trial basis. Third, the fitted model parameters enable us to study the influence of the previous action on subsequent action decisions.

### Parameter estimation and model selection

The RTs of each participant were partitioned by the four experimental conditions: action type (choice and specified trials) and repetition conditions (repetition available/absent for choice trials or repetition/non-repetition for specified trials). RT data of each condition was binned into the 0.1, 0.3, 0.5, 0.7, and 0.9 quantiles. For the choice trials, the probability of selecting each action was also calculated.

For each condition, behavioral data can be fitted by the LBA model with up to 11 parameters (the means of accumulation rates: *μ*_1_, *μ*_2_, *μ*_3_, *μ*_4_; the standard deviations of accumulation rates: *σ*_1_, *σ*_2_, *σ*_3_, *σ*_4_; the response threshold *b*; the upper limit of the starting point *c*_0_; and the non-decision time: *t*_0_). However, to accommodate differences in the observed data between conditions, one may expect that more complex models fit the data better and model parameters may change across conditions. We therefore evaluated variants of the LBA model with different parameter constrains.

First, participants may have prior bias towards a particular action and hence the accumulators may have different starting activity (i.e., *c*_0_ differs across accumulators). Second, the mean accumulation rates, the response threshold, or the non-decision time could be modulated by the experimental conditions. Third, the changes of parameters (*μ*, *b*, or *t*_0_) may apply only to the repetition alternative, rather than all the accumulators in choice trials. That is, the response of the last action trial only affects selecting the previously chosen alternative in the current trial. Finally, if one assumes that specified actions are subjected to an ordinary accumulation process irrespective to which alternative has been specified, the accumulation rates may be fixed for all accumulators in the specified action conditions.

Each model design includes a combination of the six model features above. Fig. 4 shows all 46 possible model designs that differ in model complexities and constraints. Note that the model features are not totally independent to each other. For example, whether last action trial affects only the repetition actions or all actions (i.e., the 6th feature in Fig. 4) is meaningful only if one or more model parameters (*μ*, *b*, or *t*_0_) vary between conditions. We compared all 46 meaningful models based on the presence or absence of the six model features.

For model designs that incorporate parameter changes between conditions, additional parameters were introduced to quantify the changes. The additional parameters took the form of multiples to accommodate the overall parameter changes across accumulators. For example, for a model design in which accumulation rate change only applies to the repetition alternative (e.g., the 4th design in Fig. 4), let *μ*_*a*_, *μ*_*b*_, *μ*_*c*_, *μ*_*d*_ denote the mean accumulation rates of the four accumulators in the repetition-absent condition and an addition parameter *β* denotes the accumulation rate ratio between repetition-available and repetition-absent conditions. The rates in a repetition-available trial would be *βμ*_*a*_, *μ*_*b*_, *μ*_*c*_, *μ*_*d*_, given the action *a* was selected in the last trial, or *μ*_*a*_, *βμ*_*b*_, *μ*_*c*_, *μ*_*d*_ if *b* was selected in the last trial. Modulation parameters were also use to quantify changes in response threshold or non-decision time. We used this approach based on two motivations. First, this method allowed us to quantify the effect of previous response regardless the identity of the last action (see Farrell et al., 2010; Ludwig et al., 2009). Second, this method reduced the number of free model parameters.

For each of the 46 model designs, we fitted the model to individual behavior data (i.e., a single model was used for all task conditions and parameters varied across individuals). We estimated the model prediction of RT quantiles and selection probabilities of each condition from 100,000 numerical simulations. The model parameters were determined by minimizing the likelihood ratio chi-square statistic (Ratcliff and Smith, 2004) using the Simplex search algorithm (Nedler and Mead, 1965). For each model fit, optimization procedure terminates if the minimum is obtained, or if the total number of iterations reaches a maximum of 10,000. Because the downhill simplex method may settle in a local minimum rather than the global minimum, the entire optimization procedure was repeated 20 times, and for each time started with a different set of initial parameters chosen from 100 random parameter samples which produced the best fit (Bogacz and Cohen, 2004). This procedure has been widely applied in RT modeling (Bogacz et al., 2006; Boucher et al., 2007; Dean et al., 2011). The best-fitting parameters for each model design were used to calculate the Bayesian Information Criterion (BIC) values (Schwarz, 1978), which penalize extra free parameters in favor of simpler models. The BIC values from each participant were then summed to represent the fit of the model to group data (Fig. 4). Comparing the summed BIC values of different models allowed us to identify which model provides the best description of the data across participants (Forstmann et al., 2010a; Ho et al., 2009). Nonparametric post-hoc tests were used for comparing BIC values between competing model designs.

### Estimation of expected accumulated activity (EAA)

For choice trials, the LBA model assumes three valid accumulators. Let ${\tilde{\mu }}_{W}$ be the accumulation rate of the alternative reaching the response threshold (i.e., the winner), sampled from the normal distribution *N*(*μ*_*W*_, *σ*_*W*_^2^). Let ${\tilde{\mu }}_{L1}$ and ${\tilde{\mu }}_{L2}$ be the sampled accumulation rates of the other two valid alternatives (i.e., the losers), sampled from the normal distributions *N*(*μ*_*L*1_, *σ*_*L*1_^2^) and *N*(*μ*_*L*2_, *σ*_*L*2_^2^). If the RT of the trial is *t*, the latency of the accumulation process is *t* − *t*_0_ and the expected accumulation rate of the winning alternative is:

$$E\left[{\tilde{\mu }}_{W}\right]=\frac{b-c_{0}/2}{t-t_{0}}\text{.}$$

Because the losing alternatives have not reached the threshold by time *t*, the expected values of ${\tilde{\mu }}_{L1}$ and ${\tilde{\mu }}_{L2}$ would be smaller than ${\tilde{\mu }}_{W}$. Therefore the accumulation rates of the losing alternatives have truncated normal distributions with an upper bound ${\tilde{\mu }}_{W}$, and the expected value of ${\tilde{\mu }}_{L1}$ and ${\tilde{\mu }}_{L2}$ can be calculated as:

$$\begin{array}{l} \left\{ \begin{array}{l} E\left[{\tilde{\mu }}_{L1}|{\tilde{\mu }}_{L1}<{\tilde{\mu }}_{W}\right]=\mu_{L1}-\sigma_{L1}\left[\frac{\phi \left(\frac{{\tilde{\mu }}_{W}-\mu_{L1}}{\sigma_{L1}}\right)}{\Phi \left(\frac{{\tilde{\mu }}_{W}-\mu_{L1}}{\sigma_{L1}}\right)}\right],\\ E\left[{\tilde{\mu }}_{L2}|{\tilde{\mu }}_{L2}<{\tilde{\mu }}_{W}\right]=\mu_{L2}-\sigma_{L2}\left[\frac{\phi \left(\frac{{\tilde{\mu }}_{W}-\mu_{L2}}{\sigma_{L2}}\right)}{\Phi \left(\frac{{\tilde{\mu }}_{W}-\mu_{L2}}{\sigma_{L2}}\right)}\right], \end{array}\right.\frac{-b\pm \sqrt{b^{2}-4ac}}{2a}\\ \text{where}\;\phi \left(x\right)=\frac{1}{\sqrt{2\pi }}e^{-x^{2}/2}\text{ and }\Phi \left(x\right)=\frac{1}{\sqrt{2\pi }}\int_{-\infty }^{x}e^{-x^{2}/2}dx\text{.} \end{array}$$

The EAA of each accumulator was calculated as the area under the accumulator activation prior to *t* − *t*_0_:

$$\left\{ \begin{array}{l} EAA_{W}\left(t\right)=\frac{1}{2}\left(b+c_{0}/2\right)\left(t-t_{0}\right),\\ EAA_{L1}\left(t\right)=\frac{1}{2}\left(E\left[{\tilde{\mu }}_{L1}\right]\left(t-t_{0}\right)+c_{0}/2\right)\left(t-t_{0}\right),\\ EAA_{L2}\left(t\right)=\frac{1}{2}\left(E\left[{\tilde{\mu }}_{L2}\right]\left(t-t_{0}\right)+c_{0}/2\right)\left(t-t_{0}\right)\text{.} \end{array}\right.$$

The EAA in a single choice trial is then defined by the summed activity from all the valid alternatives, given by *EAA*_*W*_ + *EAA*_*L*1_ + *EAA*_*L*2_ (Fig. 2A). For a specified action trial, we assume that only one accumulator is valid and activated, and hence EAA is equivalent to *EAA*_*W*_ (Fig. 2B).

### Data acquisition

A Siemens Tim Trio 3T scanner (Siemens Medical Systems, Germany) was used to acquire BOLD sensitive T2* weighted EPI images in sequential descending order in a rapid event-related design (TR = 2000 ms, TE = 30 ms, FA = 78°, 32 × 3 mm slices, in-plane resolution 3 × 3 mm with slice separation 0.75 mm). 1300 volumes were acquired and the first six of which were discarded to allow for steady-state magnetization. Participants also underwent high resolution magnetization prepared rapid gradient echo scanning (MP-RAGE: TR = 2250 ms, TE = 2.99 ms, FA = 9°, IT = 900 ms, 256 × 256 × 192 isotropic 1 mm voxels). Visual stimuli were presented by using Matlab 7.8 (Mathworks, Natick, MA) and the Psychtoolbox-3 (www.psychtoolbox.org), and were displayed onto a screen with a resolution of 1024 × 768 and a refresh rate of 60 Hz. Behavioral responses were acquired by using a four-button response box.

### fMRI data preprocessing

MRI data was processed using SPM8 (www.fil.ion.ucl.ac.uk/spm). fMRI data were converted from DICOM to NIFTII format, spatially realigned to the first image, and corrected for acquisition delay by sinc interpolation with references to the middle slice. The mean fMRI volume and MP-RAGE were coregistered using mutual information, and the MP-RAGE segmented and normalized to the Montreal Neurological Institute (MNI) T1 template by linear and non-linear deformations. The normalization parameters were applied to all spatiotemporally realigned functional images obtaining normalized volumes with a voxel size of 2 × 2 × 2 mm. Normalized fMRI data were smoothed with an isotropic Gaussian kernel with full-width half-maximum of 5 mm.

### fMRI data analysis

To evaluate the accumulation activity across the brain during action decisions, a first level general linear model (GLM) included three regressors. One regressor represents onsets of stimulus presentation in all task trials, and one parametric modulator represents the EAA of each trial, estimated from the fitted model parameters and the RT. The parameter beta estimates of the EAA regressor explain the effect of the trial to trial variation, which cannot be explained by the trial onset regressor. To test the differences between experimental conditions that may not be associated with the accumulation process, the model also included a third categorical regressor that contrasted the choice and specified action trials. Error trials were modeled separately. Regressors were convolved with a canonical hemodynamic response function and its first temporal derivative. Six rigid-body motion correction parameters were included as nuisance covariates. Three first-level contrast images (the effects of task, the EAA and the categorical difference between choice and specified action conditions) from each participant entered a second-level analysis (second-level ANOVA), adjusted for non-sphericity with dependence between measures and unequal variance. Statistical parametric maps were then generated for each effect of interest and corrected for multiple comparison at *p* < 0.05 (FWE corrected). To ensure that the effect of the EAA cannot be simply attributed to a linear relationship between RT and BOLD response in some brain regions (Grinband et al., 2008, 2010), we covaried out single-trial RT from the EAA (i.e., we removed the collinear component of RT from the EAA) and entered the residual of EAA as a parametric modulator in a separate first-level model (Fig. 5C).

To evaluate the influence of the previous response on the subsequent action, we included separate regressors for each condition. Linear contrasts of beta estimates were used to assess the difference of repetition availability in choice trials (repetition-available vs. repetition-absent). Regions of interests (ROIs) were defined as clusters that showed significantly stronger response in repetition-available versus repetition-absent trials (*p* < 0.05, cluster-level corrected). Individual measures of averaged effect size for each ROI were extracted using the MarsBar toolbox (http://marsbar.sourceforge.net). We then computed the Pearson's correlation coefficients between the changes in the individual model estimates from the best-fitted model (the ratio of mean accumulation rates between repetition-available and repetition-absent conditions) and the regional changes of BOLD response.

## Results

We investigated the mechanisms of voluntary action decisions in a right-hand finger-tapping task. Participants were required to make choice actions or specified actions in response to visual cues during scanning (Fig. 1A). For both types of actions, we manipulated the presence of available actions by allowing repetition responses in half of the trials, regardless of the trial types in the last action trial (Fig. 1B). Below, we first report behavioral results and the influence of previous response history on current actions. Next, we used the LBA model (Brown and Heathcote, 2008) to quantify the behavioral differences between task conditions and make inference about accumulation mechanisms during the action decision process. We then used the best-fitting model to generate parameter estimates that provided predictions about the BOLD signal, and indentify brain regions that play a role during action decision and suppression of action repetition.

### Behavioral results

**Fig. S1:**
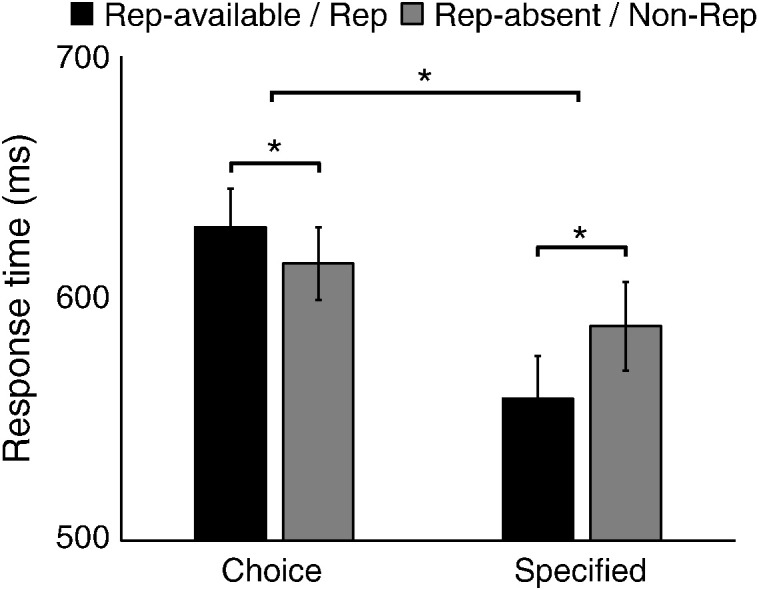
The mean RT of each condition. Error bars indicate between-subject standard errors.

**Fig. 3:**
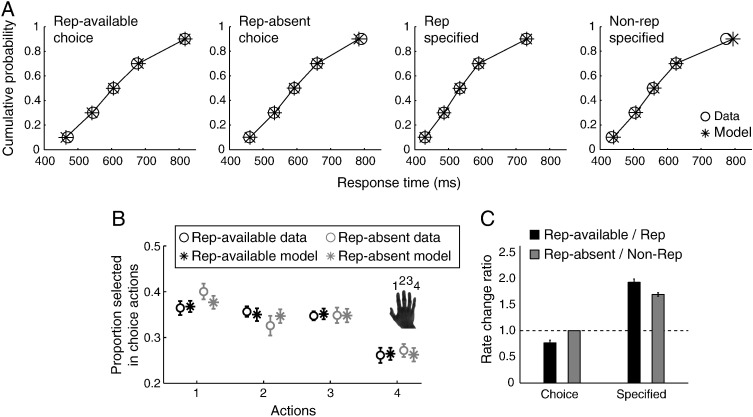
Behavioral data and model fits. (A) Best fits of the LBA model to RT quantiles in the choice and specified trials. (B) The probability of selecting each action in the choice trials. Error bars indicate between-subject standard errors. (C) Parameter estimates of the mean accumulation rate scaling for different conditions. The accumulation rate scaling for choice trials with repetition-absent served as the baseline (i.e., the ratio equals to 1).

A repeated-measures ANOVA showed longer RTs (Fig. 3A and Supplementary Fig. 1) for choice action trials than specified action trials (choice actions: 622 ± 15 ms; specified actions: 576 ± 18 ms; *F*(1,15) = 61.47, *p* < 0.00001). Response history of the last action trial had different influences on the current action, as indicated by a significant interaction between trial types (choice vs. specified) and repetition conditions (*F*(1,15) = 37.60, *p* < 0.0001). In particular, repetition facilitated RT in the specified actions (*t*(15) = 4.01, *p* < 0.001), suggesting a repetition priming effect or switch cost. In contrast, presenting the repetition alternative in choice trials slowed the action selection process (*t*(15) = − 4.12, *p* < 0.001). For choice trials, the probabilities of selecting each action (Fig. 3B) were significantly different (*F*(1,15) = 24.59, *p* < 0.001) and participants tended to suppress the repetition of the action made in the last action trial when this option was available (repetition rate: 18.9 ± 3.21%; *Z* = − 2.71, *p* < 0.01 against the 33.33% chance level, one-sample Wilcoxon signed-rank test), indicating that previous response history modulates voluntary action selection. Further, no significant difference was observed between the repetition rates of the four fingers (*F*(3,45) = 1.54, *p* = 0.22). Our results suggest that the suppression of repetition in choice actions is not due to participant's bias or preference for particular finger, but a generic mechanism for action decisions.

Occasionally participants made a commission error, responding with an invalid action (2.46 ± 0.69% of the total trials across participants). There are several possible reasons for these errors: some may be due to a lapse of attention on the task or that participants pressed a wrong button because of a muscular twitch or slip on the button box. Some may have been due to errors in action selection. However, the errors were too infrequent to incorporate meaningfully in the models, and error trials were therefore not included in subsequent behavioral and imaging analyses.

### Accumulator models for voluntary action decisions

Our behavioral data showed that the RT of action decisions was modulated by trial type and previous response history. The significant RT differences between task conditions and the variability of selection probability suggest that making voluntary action decisions is more sophisticated than a simple trigger of a random action. We conceptualized action decision in the general framework of accumulator models for decision making (Gold and Shadlen, 2007), which allowed us to unfold the decision process to investigate *which* action to select, *when* the action is initiated, and *how* action selection is influenced by previous responses. In particular, we posited that during the formation of action decisions the subjective intentions of selecting different actions are accumulated over time and competed against each other, until a response threshold is reached.

**Fig. 4:**
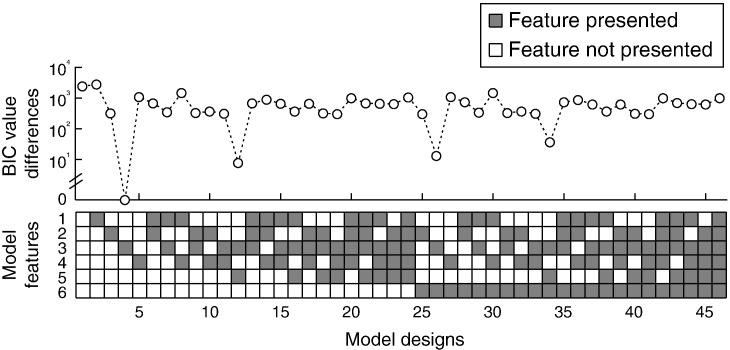
46 variants of the LBA model with different parameter constraints were fitted to individual behavioral data. The BIC values of each model design were summed across all participants to represent the fit to the group data and model complexity. The summed BIC value differences between all models and the best model are plotted against corresponding model structures which include all meaningful combinations of the six model features (as illustrated below the figure). The model features where as follows: (1) *c*_0_ differs across accumulators; (2) *b* differs across conditions; (3) the mean accumulation rate *μ* differs across conditions; (4) *t*_0_ differs across conditions; (5) the mean accumulation rates are the same for all the accumulators in specified condition; (6) the changes of the model parameters (*b*, *μ* and *t*_0_) apply to all the accumulators (rather than to the repetition alternative only). The simplest design with the best fit was given by the model with variable mean accumulation rates applied only to the repetition alternative (i.e., the 4th design in the figure.).

We modeled the accumulation mechanism by using the LBA model (Brown and Heathcote, 2008) with four independent accumulators, each representing the intention to initiate one particular action (see Fig. 1C and ‘accumulator model of action decisions’). We assumed that three accumulators are activated in a choice trial, corresponding to the three choice alternatives (Fig. 2A), whereas only one accumulator is activated in a specified trial, given the presence of only one valid action (Fig. 2B). In the simplest case, the model parameters would be constants for all conditions. However, because of the large behavioral differences between conditions, it is reasonable that some parameters may vary across conditions. For instance, the suppression of repetition in choice actions may be due to a reduction of the mean accumulation rate for the repetition alternative, or an increase of the response threshold, or an increase of the non-decision time, or a combination of different parameter changes. We considered a total of 46 LBA model designs, varying systematically in constraints on parameters across responses and across trial types, embodying differences in the mechanism of effect of prior actions, in terms of changes in accumulation rates, thresholds, non-decision times, or other model parameters. We fitted each model design on individual behavioral data and compared the model designs by the BIC values summed across all participants (see Fig. 4 and ‘parameter estimation and model selection’).

The best model design judged by the BIC values (model 4 in Fig. 4) suggested that the response threshold and non-decision time are constants (for each participant) across all experiment conditions. The behavioral differences between conditions were captured by changes only in the mean accumulation rates. Importantly, for the repetition-available condition, the rate changes applied only to the accumulator corresponding to the repetition alternative, suggesting a specific modulation of inhibition of the previous action. Compared with the simplest model (model 1 in Fig. 4), the best-BIC model had 3 additional free parameters, quantifying the change ratios in the three experimental conditions relative to the rates in the repetition-absent condition (Fig. 3C). A ratio of change smaller than 1 indicates that the mean accumulation rate is decreased compared to the repetition-absent condition, whereas a ratio larger than 1 means an increase in accumulation rate.

The best-BIC model provided an excellent quantitative estimate to the observed individual behavioral data (Figs. 3A and B). The model suggested that suppression of repetition in choice trials was described by a reduction in the mean accumulation rate of the repetition alternative relative to that in the repetition-absent condition (Z = − 3.46, *p* < 0.001, one-sample Wilcoxon signed-rank test). In contrast, specified actions were associated with increases in the mean accumulation rate (specified repetition: *Z* = 3.52, *p* < 0.001; specified non-repetition: *Z* = 3.52, *p* < 0.001; one-sample Wilcoxon signed-rank test), and the repetition priming effect in specified actions was associated with increased mean accumulation rates in repetition responses (Z = 3.309, *p* < 0.001, Wilcoxon signed-rank test).

To ensure that the model with variable accumulation rates is superior relative to our other models, we examined the next three best model designs (the 12th, 26th and 34th models in Fig. 4). Although the next three sub-optimal models included addition parameter constrains, they all shared one key feature, which was the potential for accumulation rates to vary between conditions. The best-BIC model provided significantly superior fit than the 3rd best model (model 26 in Fig. 4, *p* < 0.05, non-parametric sign test) and the 4th best model (model 34 in Fig. 4, *p* < 0.05, non-parametric sign test), but is not significantly better than the 2nd best model (model 12 in Fig. 4, *p* = 0.80, non-parametric sign test).

Further, previous studies suggest that the variable threshold in the accumulator model can provide an account of speed–accuracy tradeoff (Forstmann et al., 2008; Palmer et al., 2005), while the variable non-decision time could be associated with practice (Dutilh et al., 2009). We observed that the BIC values of the best model were significantly better than the models with variable threshold (model 3 in Fig. 4) or variable non-decision time (model 5 in Fig. 4) (*p* < 0.001, *p* < 0.0001, respectively; non-parametric sign test). Therefore we adopted the model with the best BIC values to describe behavioral data in order to inform fMRI analysis.

### fMRI results: accumulation in action decisions

The accumulation-to-threshold mechanism suggested by the LBA model provides an approximation of patterns of BOLD responses in areas involved in the accumulation process (Forstmann et al., 2010b; Ho et al., 2009). Although the relatively low spatial and temporal resolution of fMRI limits its application as a direct measure of instantaneous neural activity, the BOLD response in a single voxel may provide a proxy for the aggregate metabolic activity of large neural populations over several seconds (Rainer et al., 2001). We estimated the expected accumulated activity (EAA) of the accumulators by calculating the expected integral of all accumulators' activities prior to responses (Kayser et al., 2010; Rowe et al., 2010). The trial-by-trial estimates of EAA served as a model-based predictor to inform the analysis of fMRI data (O'Doherty et al., 2007) and identify the putative brain regions that are associated with the accumulation process in voluntary action decisions.

Using subject-specific model parameters from the best-BIC model, we estimated the EAA in each choice or specified action trial (Fig. 2 and see ‘Estimation of expected accumulated activity (EAA)’). In addition, there may exist differences between choice and specified action trials that are not due to the accumulation process, but to other fundamentally distinct processes, such as representing the target of actions or monitoring action sequences (Rowe et al., 2010). Therefore we exclusively masked the results of the EAA analysis with the categorical differences between choice and specified conditions at a threshold of *p* < 0.05 (uncorrected), reducing the likelihood that the estimation of the accumulation regions was confounded by the categorical difference between conditions.

**Fig. 5:**
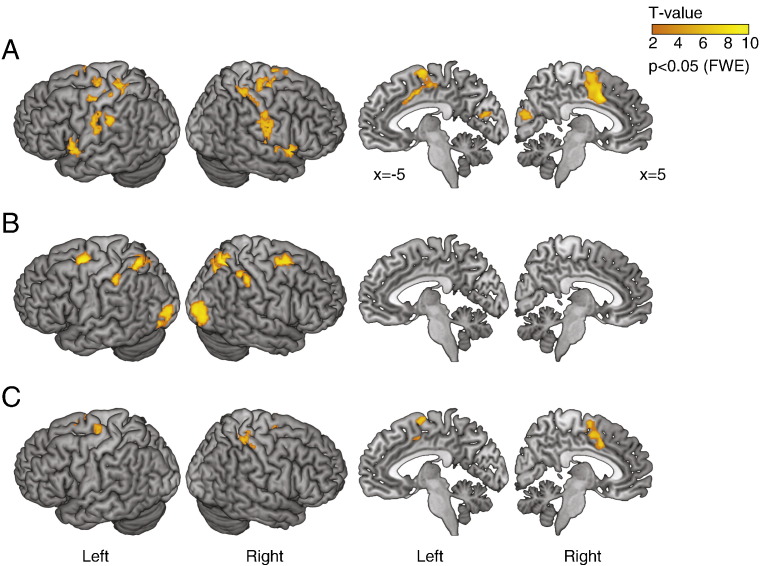
(A) Activations associated with the predicted EAA under the LBA model (*p* < 0.05, FWE corrected), exclusively masked (*p* < 0.05, uncorrected) with the categorical difference between voluntary and specified actions. Table 1 lists the cluster peaks of activations. (B) Brain regions that showed increased activity in voluntary actions than specified actions (*p* < 0.05, FWE corrected). (C) Activations associated with the predicted EAA (*p* < 0.05, FWE corrected) when the RT was covaried out from the EAA.

The EAA correlate of the accumulation process yielded clusters of significant activity (*p* < 0.05, FWE corrected) including caudal anterior cingulate cortex (ACC), supplementary motor area (SMA), pre-SMA and sensorimotor cortex (Fig. 5A and Table 1). The medial–frontal cluster extended anteriorly into the anterior cingulated cortex (*x* = − 6, *y* = 20, *z* = 30, Brodmann area 32) and ventrally along the cingulate gyrus (*x* = − 8, *y* = 18, *z* = 28). In contrast, choice actions showed enhanced fMRI responses than specified actions in the caudal prefrontal, superior and inferior parietal and occipital cortex (Fig. 5B and Table 2), consistent with previous studies using a similar task in young and older healthy participants (Rowe et al., 2010), and patients with Parkinson's disease (Hughes et al., 2010, in press).

**Fig. S2:**
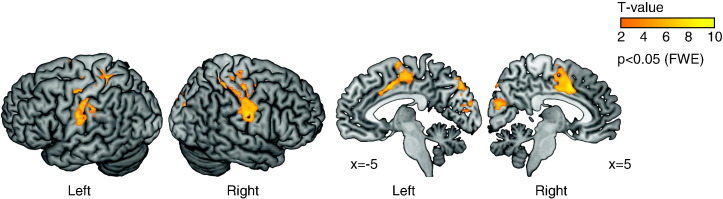
Activations associated with the predicted EAA under the simplest LBA model (model 1 in Fig. 4, p < 0.05, FWE corrected), exclusively masked (p < 0.05, uncorrected) with the categorical difference between voluntary and specified actions.

We conducted additional fMRI analysis using the EAA values estimated from the simplest model (model 1 in Fig. 4). The simplest model predicts a slightly different activation pattern compared with the best BIC model (Supplementary Fig. 2). Nevertheless the primary activation in the medial frontal cortex was not altered by using the inferior model. Since all the models assume the accumulation process, the EAA estimates may not be sensitive to the differences between model structures. Hence the behavioral data rather than the fMRI data should be used to compare between the competing models.

Further, since the EAA was calculated on the basis of single trial RT, it was possible that our results were confounded by differences in RT. In order to address this concern, we covaried out RT from the EAA in a separate analysis. Using the residual of the EAA the principal findings were not altered (Fig. 5C), although the two models showed significant differences elsewhere (particularly in the lateral–frontal cortex and lingual gyrus). These results suggest a robust activation pattern of accumulation in the medial–frontal cortex during the action decision process.

### fMRI results: suppression of repetition in choice actions

The accumulator model predicted that suppression of repetition in choice actions was mediated by changes in accumulation rates between repetition-available and repetition-absent conditions. If a brain region is involved in suppressing repetition responses, it would exhibit BOLD response differences between the two choice conditions. Therefore we first defined regions of interests (ROIs) with significant increased activation to repetition-available versus repetition-absent conditions for choice trials. BOLD signal changes in the defined ROIs were then related to the change ratio in the accumulation rates between the two choice conditions, estimated from individual behavioral data. In this way we tested a clear a priori hypothesis from the fMRI data. That is, for brain regions associated with increased activation to the presence of a repetition alternative, the extent of BOLD signal change relates to change in accumulation rate, which is in turn associated with the specific suppression of repetition in choice actions.

**Fig. 6:**
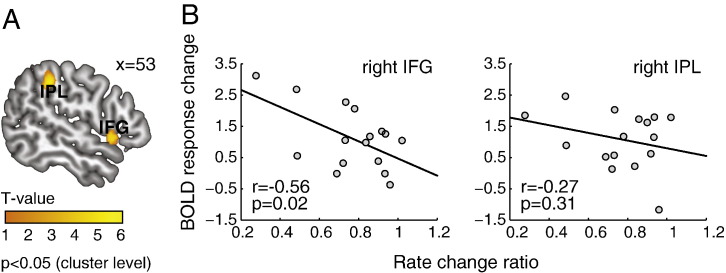
(A) Regions showing increased BOLD responses in the repetition-available condition compared with the repetition-absent trials (*p* < 0.05, cluster level corrected). (B) Scatter plots showing the inter-individual variability of the BOLD response changes in the repetition-available condition as a function of the model estimates of the accumulation rate changes (Fig. 3C). Each data point represents one participant. The solid lines indicate the linear regression.

Two regions showed increased activity in repetition-available versus repetition-absent conditions (Fig. 6A): the right inferior frontal gyrus (rIFG, Brodmann area 45: *x* = 56, *y* = 14, *z* = − 2) and the right inferior parietal lobule (rIPL, Brodmann 40: *x* = 54, *y* = − 34, *z* = 40) (*p* < 0.05, cluster-level corrected). Further, there was a significant correlation between the BOLD response change in the rIFG and level of the subject-specific reduction in mean accumulation rate of the accumulator corresponding to the action that was made previously (*R* = − 0.56, *p* < 0.05; Fig. 6B). This was not found in the rIPL (*R* = − 0.27, *p* = 0.31). The association between the rIFG activation and the change ratio in the accumulation rates cannot simply be attributed to the overall RT difference between participants, as a significant correlation was observed after mean RT was factored out of the rate change ratio (*R* = − 0.57, *p* < 0.05). Thus, participants with stronger activations in the rIFG exhibit stronger suppression of selecting a specific action on a current trial if that action has been made previously.

The relation between BOLD response change in the rIFG and the accumulation rate change ratio raises a second question: whether the rIFG activity directly relates to the raw accumulation rate. We tested this hypothesis by correlating the evoked BOLD response in the rIFG and the accumulation rate (averaged across the 4 accumulators). There was no significant correlation between the rIFG activity and the mean accumulation rate in the repetition-available condition (*R* = 0.32, *p* = 0.22) or the repetition-absent condition (*R* = − 0.06, *p* = 0.82). A further analysis showed that the correlation between the rIFG response change and the accumulation rate change ratio is significantly stronger than the correlation between the evoked BOLD response in the rIFG and the raw accumulation rates (*Z* = 2.5, *p* < 0.05; Fisher's z-transformation), indicating that the rIFG activity specifically relates to the change in accumulation rate when a repetition response is available, but does not relate to the raw accumulation rate.

Furthermore, we have evaluated the ability of two other key models to predict the BOLD response to suppression of repetition: the model with a variable threshold (the 3rd model in Fig. 4) and the model with a variable non-decision time (the 5th model in Fig. 4). No significant correlations were observed between BOLD response change and change in threshold (IFG: *R* = − 0.12, *p* = 0.67; IPL: *R* = − 0.07, *p* = 0.79) or non-decision time (IFG: *R* = 0.41, *p* = 0.11; IPL: *R* = 0.08, *p* = 0.77). We also tested the correlation between the BOLD response change in the rIFG and the accumulation rate change predicted by the 2nd best model (model 12 in Fig. 4), and did not observe significant result (*R* = 0.04, *p* = 0.87). The lack of significant correlation from the sub-optimal models provided further supports that the rIFG activity associated with the suppression of repetition in choice actions is best explained by changes in the mean accumulation rate to the repetition alternative, as suggested by the best-BIC model.

## Discussion

This study provides insight into the neural mechanisms that mediate the voluntary action decisions. By fitting optimized accumulator models of action decisions to individual behavioral data, we estimated the metabolic activity engaged in single trials and identified a decision network that was maximal in medial frontal cortex, in particular the caudal ACC and the SMA/pre-SMA. From their correlation with the predictions of the model, we suggest that these areas are implementing an accumulation-to-threshold mechanism during the formation of action decisions. Furthermore, we demonstrated that the common tendency to suppress the repetition of responses can be explained by a reduction of the accumulation rate in the accumulator models. Influence of previous responses on subsequent choice actions varies between individuals and the extent of suppression of repetition was correlated with the activation in the right IFG. Together, our findings suggest that human voluntary action decisions are mediated by complementary processes of intentional selection and inhibition.

Previous studies have linked the medial frontal cortex to response conflicts, such as volitionally controlled action (Isoda and Hikosaka, 2007; Nachev et al., 2005; Rushworth et al., 2004; Walton et al., 2004), conscious experience of action intention (Lau et al., 2004), voluntary task switching (Forstmann et al., 2006; Rushworth et al., 2002), voluntary chosen intentions (Haynes et al., 2007), error processing (Kiehl et al., 2000) and emotional conflict (Subramaniam et al., 2009). Here, we go beyond previous work by taking advantage of the computational models and single-trial inference to discern the mechanisms that mediate voluntary action generation, providing a clear link between complex behaviors and fMRI responses. We identified putative accumulator regions for action decisions mainly in the caudal ACC and the SMA/pre-SMA, which have been associated with a wide range of relevant executive functions including motor action control (Paus, 2001; Paus et al., 1993), voluntary action (Rowe et al., 2010) and attentional monitoring (Luks et al., 2002).

It has been suggested that SMA is associated with movement behavior (Fried et al., 1991), while pre-SMA activation is additionally related to a range of cognitive processes relevant for preparing motor actions, such as visuo-motor associations (Sakai et al., 1999), motor sequence planning and learning (Hikosaka et al., 1996; Isoda and Tanji, 2004; Nakamura et al., 1998), and response conflict (Ridderinkhof et al., 2004). However, cytoarchitectonic data (Inase et al., 1999), receptor-expression maps (Geyer et al., 1998), and imaging meta-analysis (Mayka et al., 2006) do not suggest a sharp distinction between SMA and pre-SMA. They suggest a rostro-caudal continuum of change in connectivity pattern and function in the medial frontal cortex, proceeding from the SMA into the pre-SMA (Nachev et al., 2007), rather than distinct clusters of subregions in supplementary motor cortex.

We compared a large set of models that systematically varied six factors, and identified the most likely of these models given the behavioral data. We cannot of course infer that our leading model is the best of all possible models, rather that it was the best within the given 46-model space. The current models have in common that the selection between valid actions and the preparation of each response are an integrated process represented by accumulation of action intentions. Voluntary actions or choices emerge from competition among the same units as mediate specified actions, even in the absence of explicit differences in reward. This is more parsimonious than a sequential model with selection–preparation–execution processes. Nevertheless, the proposed computational model includes a non-decision component which may be in relation to latency associated with action execution (Brown and Heathcote, 2008). In principle, variable action deadlines could be used to distinguish action selection/preparation from holding a prepared action for execution. Alternatively, other imaging methods with better temporal resolutions such as EEG/MEG might indicate temporal differences among the putative accumulator regions we identified.

Our findings provide novel evidence for the specific role of IFG in inhibition (Levy and Wagner, 2011). Although the right lateral–frontal cortex has been implicated in action inhibition when there are explicit, external demands to inhibit inappropriate responses (Aron et al., 2003; Chikazoe et al., 2009), here we propose that the inferior frontal cortex may also provide specific inhibition of the particular action that has been made previously, a mechanism similar to that of the inhibition of return of visual attention (Farrell et al., 2010; Klein, 2000). Such specific inhibition subserves an inherent source of suppression of repetition responses and exhibits substantial inter-subject variation. The computational modeling indicates that the mechanism of inhibition is by slowing the action accumulator, not by adjusting thresholds of the accumulator or non-decision time variance. Moreover, our imaging data identify the rIFG being the only region that not only exhibits significantly increased response in the repetition-available condition, but also correlates with subject-specific changes in accumulation rate. This specific inhibition region seems to be exogenous to the action accumulator networks in the medial frontal cortex, rather than a refractory period for action accumulators within this network. However, we cannot rule out the possibility that other brain regions within a decision network may correlate with accumulation rate changes. Analysis of the functional connectivity between the selection and inhibition regions might provide further evidence supporting a distributed mechanism for voluntary actions, but the rapid event related design is not well suited for this.

The low spatial and temporal resolutions of fMRI compared to neurophysiology do not allow us to investigate the neural mechanisms at the level of single neurons, or the time course of neural activity during decision process. Nevertheless, our methodology provides an indirect but yet sensitive tool to infer BOLD response in brain areas that are associated with accumulation processes during action decisions. Although the causal relationship between fMRI signal and neural activity is not fully understood yet (but see Logothetis and Wandell, 2004), current evidence suggests that BOLD signals are related to a time integral of activities in large neural populations (Mathiesen et al., 2000; Rainer et al., 2001). As a result, fMRI measurements combined with model-based analysis approaches (Daw et al., 2006; O'Doherty et al., 2003, 2004) could be utilized to identify putative accumulator regions. This method is not limited to the application of action decisions, as similar approaches have been successfully used in previous fMRI studies in understanding analogous accumulation processes during perceptual decisions (Heekeren et al., 2008; Ho et al., 2009; Kayser et al., 2010; Ploran et al., 2007).

In order to quantify model parameters, we have fitted the accumulator models to behavioral statistics observed from the entire experiment session. It has been widely accepted that considering the entire response time distribution, rather than the mean RT alone, can provide much richer information about the underlying cognitive processes (Ratcliff and Rouder, 1998; Ratcliff and Smith, 2004), but this relies on the assumption that the decision components (e.g., response thresholds and accumulation rate) do not systematically change across trials. We found that this assumption is not entirely true, as behavioral responses were modulated by previous response history. One limitation of our current study is that the model only captured the influence of the most recent action. Although participants were less likely to follow a specific pattern of motor sequence because different trial types were randomly intermixed, we cannot rule out that their attention or intention may vary over a long time period, which could affect the decision process, behavior and fMRI signal. It may be possible to investigate the effects of such low-frequency fluctuations on action decisions by considering the behavioral and imaging data from a longer previous response sequences (cf. Strange et al., 2005). A further potential concern is that the EAA was calculated from single trial RT and the BOLD variance explained by the EAA may be partially attributable to the RT. However, it is important to note that the EAA is estimated as the sum of accumulated activity from all valid accumulators (including accumulators corresponding to the counterfactual responses). Because of the contributions from losing alternatives, the EAA from choice action trials and RT are not necessarily linearly dependent. Further, our RT control analysis suggests that the RT only account for a small portion of the BOLD variance and our main findings of the EAA effect were not altered after RT has been covaried out in the first-level model.

Our models assume that accumulators corresponding to permitted actions are activated in a given trial, and accumulators corresponding to invalid actions do not have net accumulated activation. Without additional assumptions or modifications, the proposed model can be easily extended to any action decision tasks with *N* possible actions and *M* valid actions (*M* ≤ *N*). One may consider another mechanism that all accumulators have accumulated activations, but accumulators for invalid actions are inhibited. It is worth noting that our general model does not exclude this. Models with inhibition on invalid actions could be used to account for commission errors in which participants respond with invalid actions. However, the present study is not sensitive to quantify inhibition (were it to exist) on invalid actions by fitting error trial data, because there are only very limited number of errors (less than 10 error trials in some participants), which are insufficient to get precise RT distributions to constrain model fitting. Modeling inhibition on invalid actions is also questionable when considering a broad range of action decision tasks. For example, if the single invalid accumulator action is active but inhibited in choice trials (1 out of 4 actions is invalid), one would have to consider that the three invalid accumulators may also be inhibited in specified trials (3 out of 4 actions are invalid). However we did not observe any increased BOLD response in specified conditions than choice conditions, suggesting that increased number of invalid actions is not associated with stronger inhibition. Future studies contrasting between the current 3-choice task and a 4-choice task (i.e., no invalid action, see Hughes et al., 2010; Rowe et al., 2010) may provide further evidence to resolve the presence of inhibition on invalid actions.

Our model for action selections builds on the accumulation-to-threshold framework, which has been widely supported by studies on perceptual decisions. However the nature of the accumulated information in our model is distinct from that for perceptual decisions. Making perceptual decisions is likely to involve accumulating stochastic sensory information (Churchland et al., 2008; Roitman and Shadlen, 2002; Shadlen and Newsome, 2001). In contrast, the present study considers the voluntary action decisions that are largely internally driven or endogenously generated (Andersen and Cui, 2009; Haggard, 2008; Roskies, 2010). This type of decision should be distinguished from perceptual decisions, as the decision sometimes cannot be fully determined by external stimulus (i.e., the choice actions). Voluntary decision also differs from automatic or simple reflex actions (e.g., a knee jerk), because it requires one to choose between valid actions and control the time of execution (Brass and Haggard, 2008). We propose that during the formation of voluntary actions, the intentions (or the urge of choosing each permitted action) accumulate over time, until the intention for one action reaches a threshold. Thereby the voluntary action decision could be described by the well-established accumulation-to-threshold mechanism.

Several previous studies support our hypothesis here. EEG identifies a gradually increasing negativity beginning more than 1 s before participants are consciously aware of their pending voluntary actions, and this ‘readiness potential’ has been localized to the medial–frontal cortex (Libet et al., 1983). This progressive, preconscious activity may associate with an accumulation of intention, as direct electrical stimulation in the medial–frontal cortex elicited an experience of an urge to move a specific body part, and more-intense stimulation at the same region produced the actual body movements (Fried et al., 1991). Single-neuron recording from human medial frontal cortex provides more direct evidence (Fried et al., 2011). In their study, patients were required to make an intentional action and then report the time when they first felt the “urge to move”. Neural activity in the medial–frontal cortex (SMA/pre-SMA and ACC) exhibited a progressive increase, commencing several hundred milliseconds prior to the time of the urge to move. Further, when participants voluntarily selected their action (left or right hand), neurons contralateral to the actual action showed larger increase in firing rate than the ipsilateral neurons, indicating that an accumulator-like change in the medial–frontal cortex was associated with intentional selection between possible actions. Although the physical counterpart of subjective intention remains unclear, the neuronal process prior to actions suggests the accumulation-to-threshold mechanism for voluntary action decisions, whereas the evidence for accumulation is presumably internally driven. The same assumption has been used in studies applying the accumulation-to-threshold mechanism for other decision tasks, such as simple oculomotor responses (Bruce and Goldberg, 1985; Carpenter and Williams, 1995), value-based decisions (Krajbich et al., 2010), and time interval estimation (Simen et al., 2011). Furthermore, since we assumed that subjective intention (rather than sensory information for perceptual decisions) is accumulated during action decisions, a change in the accumulation rate should be interpreted as a change in participants' temporal preferences towards a particular action (cf. Krajbich et al., 2010), not a change of task difficulty.

Interestingly, previous studies on perceptual decisions using similar accumulator models identified accumulator regions primarily in the parietal and temporal cortices (Heekeren et al., 2008), whereas we observed markedly different brain regions in the medial frontal cortex representing accumulator pattern of activity in voluntary action decisions. This raises an intriguing possibility that the accumulation of evidence may be a generic action selection mechanism manifested in different brain regions (Gold and Shadlen, 2007), encoding either the accumulated sensory information (Gold and Shadlen, 2001) or subjective intention (Roskies, 2010), depending on the context of the task.

A challenge for future work is to extend the models to include the integration of other latent psychological processes like reward expectation. However, we suggest that the generic mechanism of accumulation-to-threshold of evidence or intentions provides a potential bridge between neurophysiology, neuroimaging and behavioral data to characterize and quantify the neural basis of complex behavior.

The following are the supplementary data related to this article.

## Figures and Tables

**Table 1:** Statistics (*p* < 0.05, FWE corrected) and peak coordinates of clusters associated with the predicted EAA reported in MNI space (mm).

Region	Hemisphere	*t*	*x*	*y*	*z*
Caudal anterior cingulate cortex	R	12.19	8	10	38
Supplementary motor area	L	8.33	− 6	− 4	68
Pre-supplementary motor area	R	8.09	8	4	50
Inferior frontal gyrus	L	7.58	− 56	10	8
Insula	R	8.02	34	28	2
Primary Motor	L	7.14	− 52	− 16	36
R	7.00	64	− 14	34
Postcentral gyrus	L	8.03	− 40	− 24	40
R	6.88	38	− 38	62
Lingual gyrus	L	7.59	− 10	− 74	16
R	6.89	14	− 64	2

**Table 2:** Statistics (*p* < 0.05, FWE corrected) and peak coordinates of clusters associated with the categorical difference between choice and specified trials are reported in MNI space (mm).

Region	Hemisphere	*t*	*x*	*y*	*Z*
Dorsal premotor	L	9.53	− 24	− 2	58
R	8.43	22	6	52
Superior parietal	L	9.56	− 20	− 68	58
R	9.57	18	− 62	56
Inferior parietal	L	8.77	− 40	− 36	40
R	7.97	− 42	− 38	50
Middle occipital gyrus	L	8.91	− 28	− 90	− 2
R	6.29	30	− 92	0
